# Development of a Sexological Ontology

**DOI:** 10.3390/s24216968

**Published:** 2024-10-30

**Authors:** Dariusz S. Radomski, Zuzanna Oscik, Ewa Dmoch-Gajzlerska, Anna Szczotka

**Affiliations:** 1 Nuclear and Medical Electronic Division, Institute of Radioelectronics and Multimedia Technology, Warsaw University of Technology, 00-665 Warsaw, Poland; 2 RadDarMed, 00-653 Warsaw, Poland; 3 Independent Researcher, 02-001 Warsaw, Poland; z.oscik@gmail.com; 4 Department of Health Science, Mazovia Academy of Applied Science, 08-110 Siedlce, Poland; edg@misal.pl; 5 Warsaw Institute of Sexology and Psychotherapy, 00-508 Warsaw, Poland; annaa.szczotka@gmail.com

**Keywords:** ontolology, sexual reactions, physiological measurements, mathematical modeling

## Abstract

This study aimed to show what role biomedical engineering can play in sexual health. A new concept of sexological ontology, an essential tool for building evidence-based models of sexual health, is proposed. This ontology should be based on properly validated mathematical models of sexual reactions identified using reliable measurements of physiological signals. This paper presents a review of the recommended measurement methods. Moreover, a general human sexual reaction model based on dynamic systems built at different levels of time × space × detail is presented, and the actual used modeling approaches are reviewed, referring to the introduced model. Lastly, examples of devices and computer programs designed for sexual therapy are described, indicating the need for legal regulation of their manufacturing, similar to that for other medical devices.

## 1. Introduction

Sexual health is an essential component of human health and well-being. The World Health Organization defines sexual health as “a state of physical, emotional, mental and social well-being about sexuality; it is not merely the absence of disease, dysfunction or infirmity. Sexual health requires a positive and respectful approach to sexuality and sexual relationships and the possibility of having pleasurable and safe sexual experiences free of coercion, discrimination, and violence. For sexual health to be attained and maintained, the sexual rights of all people must be respected, protected, and fulfilled” [1]. This can be achieved, in part, based on work in the following fields:

- Research on the prevention and control of sexually transmissible infections, including HIV.
- The prevention and management of cancers of the reproductive system.
- Education, counseling, and care related to sexuality, sexual identity, and sexual relationships.
- Sexual function and psychosexual counseling.

Moreover, the World Association of Sexual Health published the Declaration on Sexual Pleasure. This declaration states that sexual pleasure is the physical and/or psychological satisfaction and enjoyment derived from shared or solitary erotic experiences, including thoughts, fantasies, dreams, emotions, and feelings [1]. Unfortunately, historically, human sexuality and reproduction were treated more like a mystery than a part of human physiology. As a result, knowledge about the sexual reactions of people usually comes from cultural messages, and it is limited by moral rules existing in a given society. Sexology is one of the youngest disciplines of medicine. Its beginning dates back to 1970, when Masters and Johnson presented the first model of male and female sexual reactions based on observational and experimental studies [2]. However, unlike other medical fields, clinical sexology is not yet based on evidence. Moreover, definitions of basic terms such as libido, sexual drive, sexual arousal, and orgasm, as well as the understanding of the sexual reaction mechanism, vary between scientists and clinicians. Therefore, we consider the need to build an ontology of sexology.

The goal of this study was to present a new concept of sexological ontology. This ontology must be built on properly validated models describing human sexual reactions at different resolution levels, taking the biopsychosocial character of human sexuality into account. Models should be identified based on reliable and valid measurements. Therefore, we also present the measuring and modeling methods applied in sexology, which can be useful in sexological ontology building. Moreover, we discuss the prospects for the development of devices that are consistent with evidence-based sexology, and those that can aid in sex therapy.

## 2. Sexological Ontology

In information science, an ontology is a way of showing the properties of a subject area and how they are related by defining a set of terms and relational expressions that represent the entities in that subject area. An ontology of a given knowledge branch helps researchers and clinicians to communicate among themselves. In medicine, an ontology also enables the creation of standards for disease diagnosis and management. The best-known biomedical ontologies are the anatomical ontology, gene ontology, and radiological ontology [3,4].

**Figure 1:**
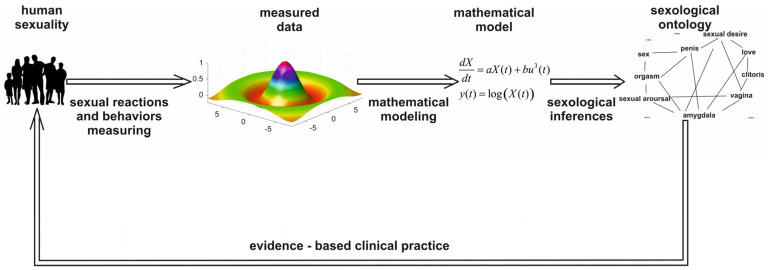
Measurements and mathematical models used as tools to build human sexuality ontology.

In our opinion, the most important challenge in sexology is building an ontology containing unambiguously defined terms and their relationships. Such an ontology would provide a shared understanding and language for communication between partners, specialists, and researchers, which would overcome the semantic heterogeneity still present in sexology. For instance, there is a semantic overlap between the terms libido, sexual desire, and sexual arousal. Moreover, no definition of orgasm is widely accepted. Clinical entries in the International Classification of Diseases, 11th Revision (ICD-11), and the Diagnostic and Statistical Manual of Mental Disorders, Fifth Edition (DSM-V), classification systems are also significantly different. These facts strongly affect diagnoses of sexual disorders and methods of their treatment. A sexological ontology could therefore help to establish conformity between the DSM and ICD classifications of sexual dysfunctions and disorders. Last but not least, ontology enables the building of computer systems that aid in medical and psychological diagnoses and patient health management, contribute to the Medical Subject Headings (MeSH) thesaurus devoted to the effective searching of publication databases (e.g., PubMed), and maintain the coherence of patient data within health information systems. An ontology is also necessary for developing modern digital sexual health. Therefore, we propose a method of sexological ontology building, as shown in Figure 1.

The first step in the proposed process is to collect data representing the studied attributes of human sexuality. Unfortunately, many theories describing sexual reactions and behaviors are formulated based only on clinical experience, and have narrative forms. For example, Basson’s model of female sexual reactions [5] is a theoretical model expressed by a directed cyclic graph composed of the following mutually dependent latent variables: emotional intimacy, sexual stimuli, sexual arousal, sexual desire, growing excitement, and emotional and physical satisfaction. In this situation, measured data are unknown, so these theories cannot be validated in replicated studies.

We believe that the sexological ontology should be built based on properly validated mathematical models because such models enable the filtering of evidence-based theories away from clinicians’ opinions and speculations. The accessible modeling methodology is also discussed.

The interpretation of these models using sexological terms would lead to the development of theories for explaining the psychophysiology or pathology of sexual reactions and behaviors. From a biomedical engineering point of view, these theories should be used as a scientific basis for the development of medical devices supporting sexual therapy and rehabilitation. In the next sections, we present our recommendations for reliable and valid measurement techniques and modeling methods to build a sexological ontology. Lastly, we also show examples of sexual devices that can be effectively used in clinical sexology.

## 3. Measuring Human Sexuality

Human sexuality seems to be the most complex life process. By complexity, we mean a combination of the following three elements: the number of components, the pattern of the connections (which components are related), and the nature of these connections (the nonlinearity degrees of the relationships). Indeed, this process depends on many interplaying social, psychological, and biological factors.

Psychological and sociological factors are usually measured using questionnaires created according to psychometric/sociometric methodologies, e.g., the Female Sexual Function Index. However, measurement theory is also helpful in this area. For example, in sexology, one often deals with unobserved variables. In such a situation, we can measure an unobserved variable by introducing an *instrumental variable*, i.e., a newly observed variable that is highly correlated with a given unobserved variable, and uncorrelated with the remaining variables and random factors. For instance, when assessing the circumference of a penis, the question “What is the size of your penis?” does not represent an instrumental variable, because size is correlated not only with penile girth but also with penile length. Another example of the application of measurement theory to construct a questionnaire is item response theory. This allows for the differentiation of the significance of individual items in a psychometric test. This method was used by Fino et al. to develop the Sexual Risk Behavior Scale [6].

From a biomedical engineering point of view, biological factors are the most interesting because different types of sensors must be used to measure them. These measurements are needed to develop a physiological model of sexual reactions. It is worth mentioning that sexual activity involves all human physiological systems. Thus, we can distinguish three groups of physiological measurements that are useful for the development of sexual reaction models, as follows:

- Measurements of genital responses to sexual stimulation;
- Measurements of peripheral system responses to sexual stimulation;
- Measurements of brain responses to sexual stimulation.

The measurement techniques for genital sexual responses strictly depend on the phenotypic sex. For men, plethysmography remains the recommended technique; it measures the increase in penile volume during the presentation of sexual stimuli. There are two procedures for conducting penile plethysmography. The first is volumetric plethysmography, which measures air pressure changes in a small cylinder and latex cuff enclosing the penile body. This is an undirected method. The second is circumference plethysmography, which measures changes in penile girth using tensometers. An interesting comparison of these methods was carried out by Kuban et al. [7]. They showed that volumetric plethysmography had better measurement sensitivity than the circumferential method.

However, the latter showed significant penile girth reduction during the extended phase of the sexual response. We suggest that this feature may be useful for examining patients with erectile dysfunction resulting from a blood flow imbalance in the penile vessels. In these patients, prolongation of the penis is preserved. Penile plethysmography was mainly introduced to forensic sexology to test potential offenders’ sexual responses to pedophilic visual stimuli. Some clinicians question its usefulness in criminal proceedings, mainly because the high prevalence of erectile dysfunction may give false negative results concerning circumferential plethysmography. The next problem is to determine an unbiased index and a cut-off value thereof that can indicate pedophilia. There are different indices, e.g., those based on the z-score transformation of the plethysmographic curve, the difference in or ratio of the response value with a maximal stimulus (a naked child) and a neutral stimulus, and an area under the response curve estimating a global penile reaction [7]. We believe that all of these indices are highly mutually correlated; however, they are unfortunately also biased by at least two factors. Firstly, habituation bias is well-known in behavioral tests, according to which the next response to the same stimulus is always lower. The second affecting factor is stress associated with the test, which can inhibit blood flow to the penis and consequently reduce its circumference. Thus, the measurement reproducibility is also low. However, we offer ways to avoid these drawbacks. Habituation to adverse effects may be limited by introducing pseudorandom sequences of visual stimuli in future tests. It seems reasonable to measure only penile prolongation in the first several seconds of the sexual response because it usually weakly depends on mental inhibition.

**Figure 2:**
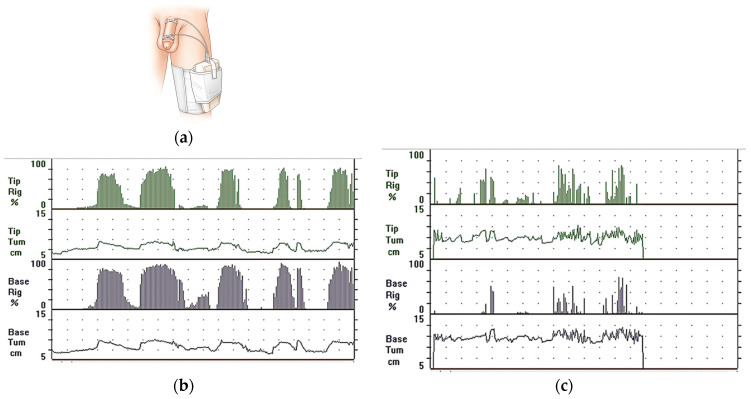
RigiScan for evaluation of nocturnal penile erections: (**a**) measuring method; (**b**) recording in a 36-year-old man with psychogenic erectile dysfunction. Five well-defined erectile events are recorded with more than 10 min duration and rigidity at the tip of the penis more than 70% (in 4/5 events). This is a “classic normal” recording in psychogenic cases.; (**c**) recording in a 54-year-old man with mixed vasculogenic erectile dysfunction. This is a “classic abnormal” recording in vasculogenic cases [8].

Penile plethysmography seems to be effective in identifying nocturnal penile erections during the REM phase of sleep based on EEG signals. In this examination, the influence of psychogenic factors is reduced. Thus, nocturnal plethysmography can help to differentiate between the psychogenic and biological causes of a patient’s erectile dysfunction. For that purpose, RigiScan, shown in Figure 2, is proposed [8,9].

Edgar et al. [10] recently presented a novel, more comfortable tool, combining mechanical sensors with temperature sensors, assuming that blood flow to the penile arterioles increases the temperature of the penis. This measuring system may be useful for identifying the vascular causes of spontaneous nocturnal erectile disorders, as opposed to disorders resulting from injuries of the corpus cavernosum [10].

**Figure 3:**
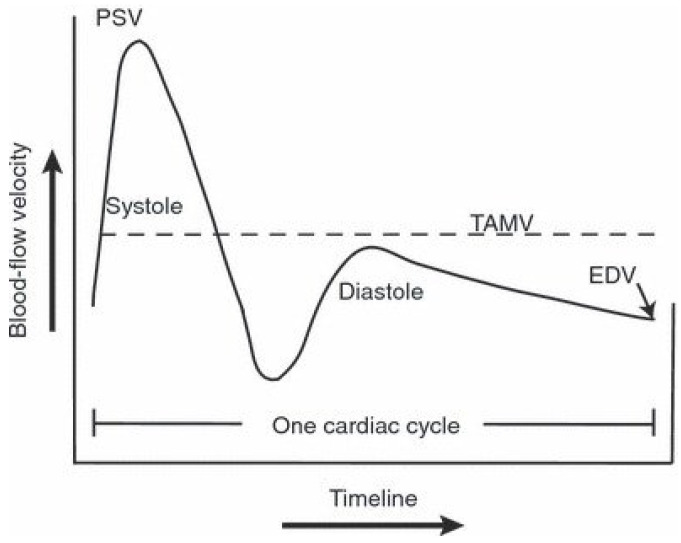
Biophysical interpretation of the parameters used to assess blood flow in clitoral or penile vessels.

Plethysmography cannot be immediately applied to women because of the anatomical conditions; thus, different versions of this method have been proposed to measure the arousal reactions of the female body. These methods can be classified into the following categories: genital blood inflow assessment, vaginal mechanic assessment, lubrication assessment, muscular assessment, and peripheral neural response assessment. The most reliable method is light plethysmography. A probe is inserted into the vagina, and it is equipped with sources of infrared or laser light and sensors measuring the reflections from the vaginal arterioles. The received signal is called the vaginal amplitude pulse (VAP), and it has two components. The constant component measures the blood volume in the vaginal arterioles. The alternating component quantifies the pulses of the vaginal walls caused by the cardiac stroke volume. A spectral analysis of the VAP signal performed by Rafiee et al. showed that, in sexual arousal, the power and frequency of the respiratory and cardiac components were higher than in the case of ambivalent stimuli [11]. Moreover, the lowest frequencies of the VAP signal were dampened because of vaginal prolongation and rigidity. Alternative methods applied to measure vaginal blood flow include oxygenation measurements, Doppler ultrasound methods, and thermographic methods. For example, Doppler ultrasound measurements are increasingly commonly applied to assess blood flow in the clitoral vessels using parameters such as the pulsatility index (PI), resistance index (RI), peak systolic velocity (PSV), end-diastolic velocity (EDV), and time-averaged mean velocity (TAMV). The biophysical interpretation of these parameters is shown in Figure 3.

Fernandez Perez et al. proposed to use the blood volume flow rate in the clitoral arteries as a product of TAMV, and the cross-sectional area of the examined artery [12]. Indeed, the rigidity of the clitoris, as well as that of the penis, depends on the blood volume, but not blood flow. Thus, this parameter is more correlated with female sexual arousal.

**Figure 4:**
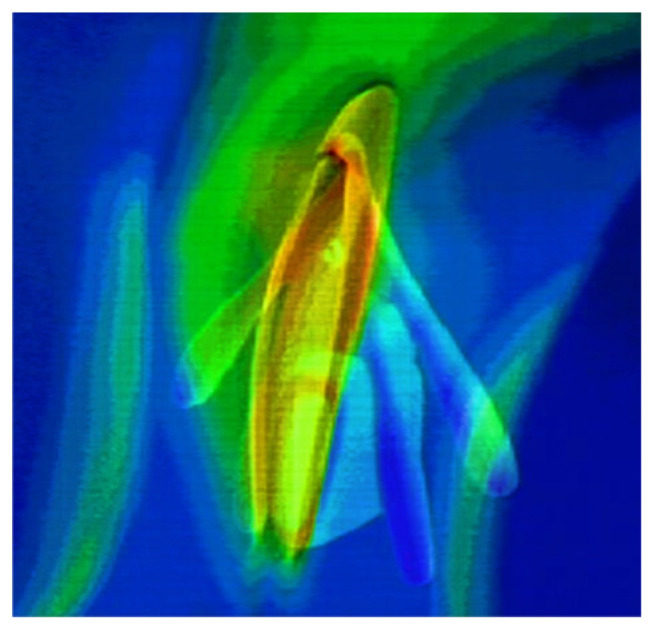
The temperature distribution of the vulva obtained using thermography during sexual arousal. The green and red colors represent higher temperatures. (https://www.sciencephoto.com/media/638333/view/female-genitals-thermogram-and-animation, accessed on 16 August 2024).

Doppler ultrasound or laser Doppler instruments measure only the strictly local genital response to sexual stimuli. Thermography is a more global technique used in the evaluation of female sexology. Ines Tavares et al. published an excellent review of infrared thermography applied to the human psychophysiology of sexual reactions [13]. Furthermore, Figure 4 presents the temperature distribution of the vulva during sexual arousal; in it, we can see an increase in the temperature of the labia minora and clitoris.

Based on this review, we conclude that the dynamics of genital temperature variability highly correlate with VAP signals. However, this method is more accepted by men and women because it is touchless.

Interestingly, from a clinical point of view, in women and men with sexual arousal disorder, compared to healthy people, there is no correlation between an increase in temperature and an increase in subjective arousal. Moreover, in women with sexual pain penetration disorder, the dynamics of temperature increase are lower than in healthy women. However, this does not distinguish between mental or somatic etiological mechanisms. We believe that thermography used with visual and touch stimuli (mechanical or electrical stimuli) can differentiate these mechanisms. In psychogenic arousal disorders, the thermic genital reaction may occur as a spinal cord reflex despite subjective arousal. On the contrary, patients with somatic etiology feel subjective arousal without genital thermic reactions.

**Figure 5:**
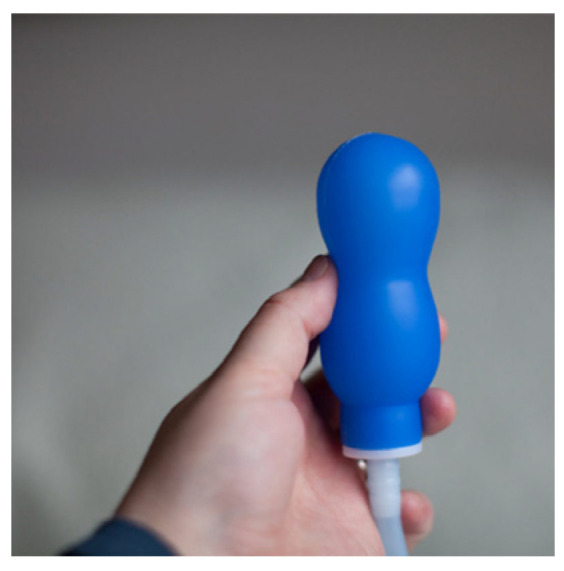
The vaginal probe pressure 1 balloon Epi-no Delphine Plus (https://www.epino.de/en/epi-no-delphine-plus.html accessed on 23 October 2024).

Another method used for the assessment of female sexual responses measures the mechanical contractions of the vaginal walls and pelvic floor muscles. The most modern vaginal probe has several tensometric or pressure sensors, which measure the mechanical strain of the vaginal walls and/or pelvic floor muscles [14]. The sensor shown in Figure 5 may be applied in the vagina, as well as in the rectum. This method is resilient to body movements, so it is dedicated to studies of vagino–rectal contractions during orgasm, when the whole pelvis makes significant movements. Measurements have shown that the best genital sign of a female orgasm is rectum contractions with a frequency equal to 8–13 Hz [15]. Moreover, these contractions cannot be voluntarily evoked during fake orgasms.

Alternative methods used for the genital monitoring of orgasm include electrovaginography and electromyography of the pelvic floor muscles. The first one involves placing electrodes on the anterior vaginal wall. Such measurements have shown that there is a pacemaker localized around the G-spot. The amplitude and frequency of the pacemaker’s bioelectrical activity depend proportionally on vaginal volumetric dilation [16]. This indirectly suggests that penile circumference may be an important biomechanical factor in improving the achievement of female orgasm. Perhaps the pacemaker also induces vaginal contractions during fetal head passage. Clinically, electrovaginography can be used to evaluate the functional results of G-spot surgical correction [16].

Electromyography of the pelvic floor muscles is a similar technique, where outer surface electrodes are attached to the vulvar skin, or transvaginal electrodes are applied. An analysis of the pelvic floor EMG signals in both the time and frequency domains has confirmed that orgasm is associated with high-amplitude and -frequency activities of these muscles. Clinically, surface EMG is useful for diagnosing vaginismus and testing the contractions of the pelvic muscles in response to touch. These measurements are also employed for biofeedback in behavioral vaginismus therapy [17], as they increase treatment effectiveness.

**Figure 6:**
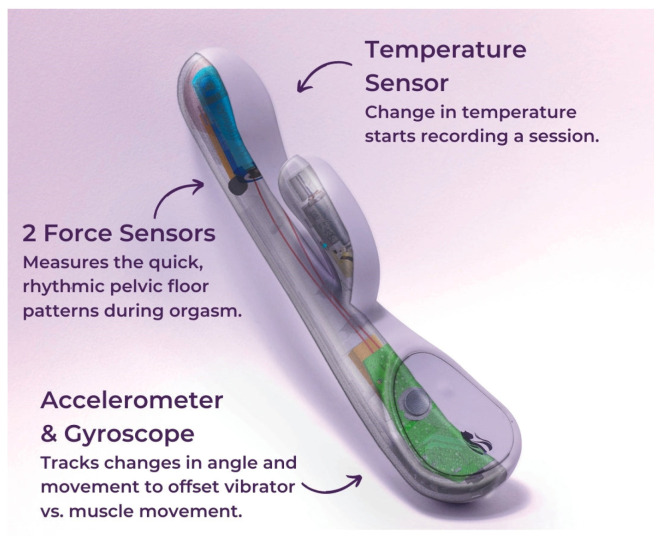
Smart vibrator equipped with three types of sensors to measure the sexual response of female genital organs (https://lioness.io/products/the-lioness-vibrator, accessed on 16 August 2024).

In our opinion, a very useful technique may be a new, smart vibrator produced by Lioness. It is equipped with force sensors measuring the contractile patterns of the pelvic floor muscles, temperature sensors measuring the temperature in the vagina, and accelerometer/gyroscope sensors measuring the vibrator’s movements and vibrations (Figure 6). However, we stress that the use of this device as a scientific instrument requires an estimation of the measurement reliability and validity.

**Figure 7:**
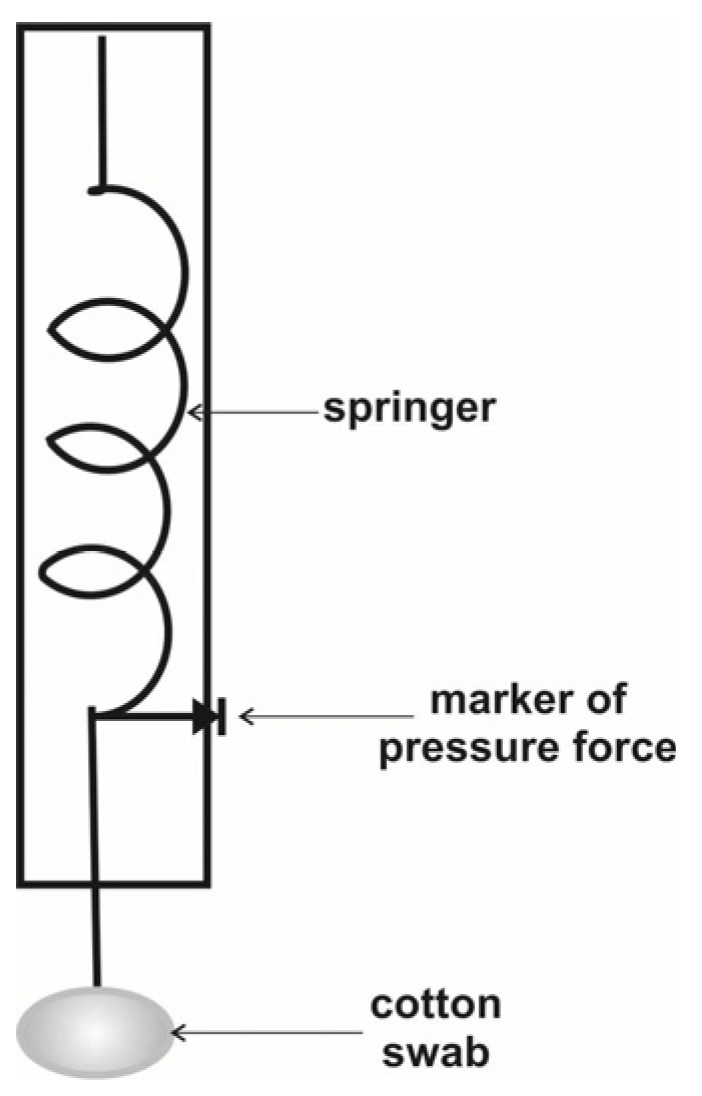
Schema of a vulvagesiometer for vulvar pain threshold assessment.

In pathological situations, the genital response is often weakened by urogenital pain syndrome. Thus, it may be necessary to measure the pain threshold in the vulvar area. For this purpose, the vulvalgesiometer, elaborated by Caroline Pukall et al. [18], can be applied. The vulvalgesiometer is a simple mechanical device consisting of cylindrical, hand-held, pen-like devices containing springs with varying compression rates. This set exerts a wide range of forces, from 3 g to 1 kg, with an excellent representation of twenty-four levels between these two limits. At the bottom of each device is a plastic piece holding a standard-sized cotton swab. The cotton swab tip (approximately 4.5 mm in diameter) is placed perpendicularly to the skin area being tested. It is then manually pushed down onto this area until the top of the inner white plastic piece reaches a marked level on the cylindrical casing, exerting a pre-specified level of force. The cotton swab is easily disposed of and replaced, removing the need for lengthy sterilization processes [17]. This is an algometer adapted for the vulva. A scheme of the vulvagesiometer is shown in Figure 7. We stress that the pain and touch threshold values could confound or modify the relationship between erotic touch stimuli and genital responses.

Sexual responses involve all physiological systems because they induct the parasympathetic or sympathetic nervous system. Thus, the second group of physiological measurements capture data on the sexual response of the sympathetic or parasympathetic nervous system. Typically, the following physiological parameters are measured: blood pressure, heart rate, skin electrical conductance, and pupil area [19]. The values of these measurements increase as sexual arousal approaches maximal intensity [20]. The newest physiological parameter observed in sexology is heart rate variability. There is an inverted U-shaped relationship between heart rate variability and sexual reactions, which confirms that a balance between the parasympathetic and sympathetic nervous systems is necessary to achieve an orgasm [21]. Additionally, clinically, low heart variability coexists with sexual dysfunctions because the sympathetic nervous system has advantages over the parasympathetic one. This can help diagnose a given dysfunction’s psychogenic etiology [22].

The third group contains the measurement techniques focused on the brain’s responses to sexual stimulation. They have recently been used to examine the structural and functional role of the brain in the control of sexual reactions. MRI enables us to visualize and measure the volume of the cerebral structure in relation to a given sexual dysfunction. For example, Atmaca et al. found that the mean right amygdala volume of patients with vaginismus was smaller than that of healthy controls. Similarly, the mean left and right hippocampus volumes were also smaller [23]. Such measures help us to better understand the pathophysiology of vaginismus.

Thus, fMRI and EEG are technical tools that enable us to measure the dynamic activity of the brain during sexual reactions, as well as to visualize neurophysiological representations of sexual emotions such as love. Despite several methodological issues, fMRI allows for the identification of the areas that are activated with sexual activity, as listed in Table 1 [24]. Moreover, with fMRI, we are trying to understand the phenomenon of romantic love. A study performed by Wang et al. shows that romantic love is associated with lower overall brain functional segregation and an enhancement of socio-emotional processing [25].

Positron emission tomography is relatively rarely applied in sex studies. However, by using PET, Huynh et al. observed increased blood flow to the pituitary gland during orgasm [26]. This confirms the clinical results of the elevated concentrations of prolactin and oxytocin after orgasm, particularly in women [27].

Another significant technique rarely used in sexological studies of the brain is electroencephalography (EEG). Through the use of EEG, we know that sexual arousal inhibits bioelectrical activities of the prefrontal cortex. Additionally, the desynchronization of the hemispheres is a normal phenomenon accompanying orgasm [28]. Moreover, when approaching orgasm, there is an increase in alpha waves in EEG signals. In the orgasmic period, EEG signals look like epileptic seizures [29]. This supports the hypothesis that orgasm can be treated as a type of meditation that is equivalent to a tantric understanding of human sexuality. Therefore, we believe that the fusion of spatial EEG maps and fMRI maps can provide valuable information for understanding the neurophysiology of the sexual response cycle.

To summarize, the most important goal of measurement techniques is to provide possibly objective information about physiological mechanisms controlling sexual reactions and behaviors. Compared to other medical disciplines, the signal analysis methods applied in sexology are still poor. In particular, there is a lack of time–frequency representations of these signals, which would allow for studying the time dependency between the genital, muscular, cardiovascular, hormonal, and neural components of the sexual response to stimuli. The measured values of a human system’s psychic and biological states are employed to identify models describing mechanisms of human sexuality.

## 4. Identification of Human Sexuality Models

The next step in sexological ontology building is to identify a model that describes the mechanism of human sexuality. Modeling has the following two purposes:

- A scientific purpose—to understand the biopsychosocial mechanisms that govern healthy or pathological sexual reactions and behaviors;
- A clinical purpose—to identify the sexual health of a patient in order to personalize their psychotherapy or medical treatments.

We strongly emphasize that mathematical models are the only tools intended to verify the agreement between conceptual sexual mechanisms and clinical or experimental data.

Formally, using a model of a sexual reaction, we use the following abstract structure $M=\left\{A,V,R,\left\|\cdot \right\|\right\}$ where

A is a set of assumptions underlying the given model;

V is a set of independent, confounding, and dependent variables considered in the given model;

R is a set of relationships among the model variables;

$\left\|\cdot \right\|$ is a norm expressing how much the given model fits the measured variables or mimics the studied mechanism. Usually, the norm is a *p*-value calculated from the distribution of test statistics or information-based criteria. In a more sophisticated case, the norm could be an error between the sexual reaction predicted by the given model and the real sexual reaction.

In our opinion, this is the most generalized definition of a model. Most of them neglect the assumption set, and this leads to unfounded conclusions being drawn.

**Figure 8:**
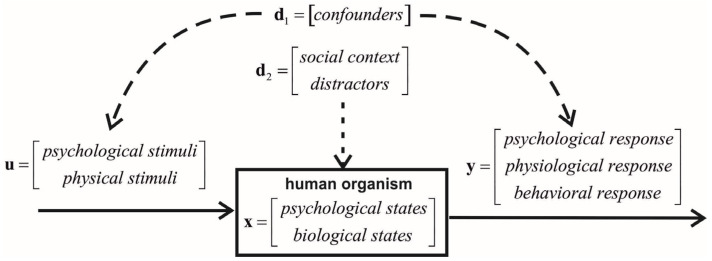
Biocybernetic view of human sexual reactions.

We propose looking at human sexual reactions from a biocybernetic perspective. Then, the mechanism of these reactions should be perceived as a multivariate, complex dynamical system, as shown in Figure 8.

It is obvious that the target model should have the following form of state equations:

(1) $$\begin{array}{l} \frac{dx(t)}{dt}=f\left(x(t),u(t),d_{2}(t)\right)+\varepsilon (t)\\ y=g\left(x(t),d_{2}(t)\right) \end{array}$$

where

- u(t) is a vector representing measurable or latent psychological (e.g., cognitive, emotional, and imaginary) and physical sexual stimuli.
- x(t) is a vector of the measurable or latent state variables describing the psychological and biological states of a human organism influenced by sexual stimuli (e.g., emotional arousal, blood pressure/flow, heart rate, hormone concentrations, and brain activities).
- $d(t)={\left[\begin{array}{cc} d_{2}(t) & d_{1}(t) \end{array}\right]}^{T}$ expresses measurable or latent disturbances affecting the sexual reaction. This vector should consist of two subvectors. Subvector $d_{2}(t)$ portrays the distractors or social contexts that affect the psychological or physiological state variables (e.g., distress and anxiety). Subvector $d_{1}(t)$ contains confounders (e.g., age, sexual orientation, and sexual and gender identity). Let us remember that a confounder is a factor that is correlated with the sexual stimuli and sexual responses but does not causally affect any state variable. The confounders can be identified using *d-separation criteria* [30].
- y(t) is the output vector composed of the measurable or latent psychological (e.g., satisfaction), physiological (e.g., penile or clitoris erection), and behavioral (e.g., involuntary and pelvic movement) sexual responses.
- ε(t) is a random vector modeling the inter-individual variability in a given population. Its probability distribution must mimic the real variability, which is not always Gaussian.
- f(·) is a vector of the functionals describing the causal relations between the sexual stimuli, the psychophysiological states of a human organism, and the disturbances.
- g(·) is a vector of the functionals transforming the psychophysiological states of a human orgasm into the observed.

Because of system complexity, the functionals f(·), g(·) usually cannot be derived from mass, momentum, or energy conservation principles. Their form must be identified based on observational or experimental data.

The high dimensionality of the model vectors prevents the modeling of a complete, biopsychosocial system of the human sexual reaction because of the data resource and computational limitations. In our opinion, the modeling process requires the following steps:

- The definition of the model scope. By the scope of a model, we denote the range of phenomena that the model describes.
- The abstraction of the studied part of the sexual reaction mechanism from the whole system.
- The definition of the granularity of the studied process. Granularity is the number of elements that compose the process. For example, at the lowest granularity level, the mechanism of sexual reactions can be represented as the following sequence of four elements: sexual desire -> sexual arousal -> orgasm -> relaxation.

**Figure 9:**
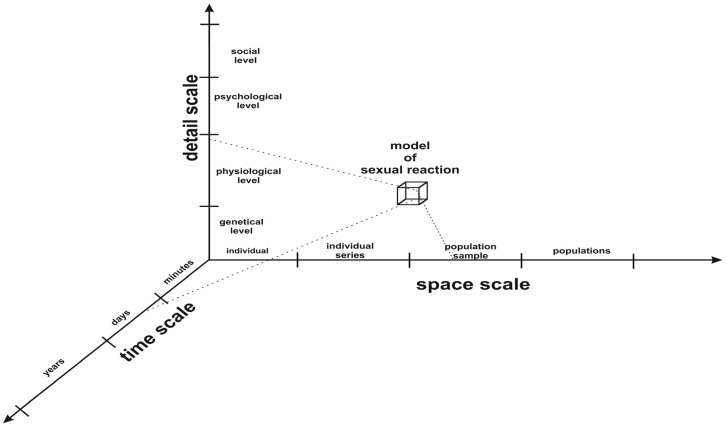
The model time × space × detail resolution levels of sexual reaction models.

The above-mentioned steps strictly determine the model time × space × detail resolution level, as shown in Figure 9. The model resolution also defines the scope of the model-based scientific or clinical inferences.

However, the proposed system theory-based approach is almost never applied in sexology. Most models have a low resolution. Additionally, verbal models are usually used, where the relationship between the psychic and biological factors controlling human sexuality is represented by verbal descriptions built based on the clinical experience of the model creator. An example of such a model is the model of a female sexual reaction proposed by Basson and presented as a finite automaton, assuming a low granularity level [5]. Basson arbitrarily singled out certain sexual states and determined the transitions between them. This model is built based on clinical, unsystematic observations, and has never been validated. Thus, other authors cite and discuss different versions of the model.

As in other medical disciplines, most models are used to identify a potential association between a dichotomous independent variable (i.e., stimulus vs. non-stimulus and personality trait occurrence vs. non-occurrence) and a dependent variable representing a mental or physiological response accompanying sexual reactions. Identifying these models often involves testing hypotheses by comparing a control to a group of subjects or the experimental conditions studied. Such models are obviously nonparametric because they do not identify or specify any form of relations between the studied variables, although the probabilistic distribution family of these variables can be known. Moreover, they do not control associations for any confounding variable.

Regression models are a class of more advanced models applied in sexology. Modern regression models, such as mixed random effect models and multilevel models, have recently been applied. They allow for the inter- and intra-variability of sexual reactions to be taken into account. However, their identification and interpretation require much knowledge about modeling methodology. For instance, such models have been used to identify the relationship between genital sexual reactions and subjective arousal in women depending on different factors, such as the level of anxiety, stress, partner intimacy, and communication [31].

The latest trend in human sexuality modeling is to employ machine learning methods. These explorative models are particularly useful for complex systems with poor a priori knowledge about the phenomenon under analysis. Human sexuality fulfills that requirement. Machine learning-based models enable the identification of nonlinear relations between large sets of variables. However, they discover knowledge only based on the data harvested. Thus, researchers must be careful in model interpretation because some of those relationships cannot be interpreted from the point of view of psychophysiology. The results obtained using machine learning-based models require confirmation in more restrictive observational or experimental studies. However, such models may effectively predict sexual reactions and behaviors. The most interesting application of machine learning-based models was for the prediction of sexual satisfaction and sexual desire. Both were identified based on a machine learning algorithm called “random forest”. The first model contained 91 variables as potential predictors, which were reduced to 21 in the model identification procedure. This model explained about 45% of the female sexual desire variance and 25% of the male sexual desire variance [32]. The goal of the second model was to predict partners’ decisions about their relationships after speed dating based on their own preferences measured before speed dating. Although the model contained 200 variables, it explained only 20% of the decision variance. This model confirmed that partners’ desire develops during their interactions [32]. Similar results were obtained in a longitudinal study. The initial intra- and inter-assessments of the partners studied explained only 18% of the variance of relation satisfaction in the time endpoint. Finally, relationship quality change (i.e., increases or decreases in relationship quality over the course of a study) was largely unpredictable from any combination of self-report variables [33]. Although these models do not explain any psychosexual mechanism in detail, they support the statement that a partner’s psychosexual relationship is an adaptive, nonlinear dynamic system affected by numerous confounders. Moreover, it seems that it is not a globally controlled, but almost globally observable system.

Hypothesis testing-based models and regression models do not provide any insight into the structure of (causal) relations between study variables. Knowledge of this structure is necessary to formulate state equations. Therefore, we recommend using structural equation models (SEMs) or Bayesian nets more frequently. The former allow for the identification of linear relations between variables, and the latter also allow for the identification of nonlinear dependencies.

**Figure 10:**
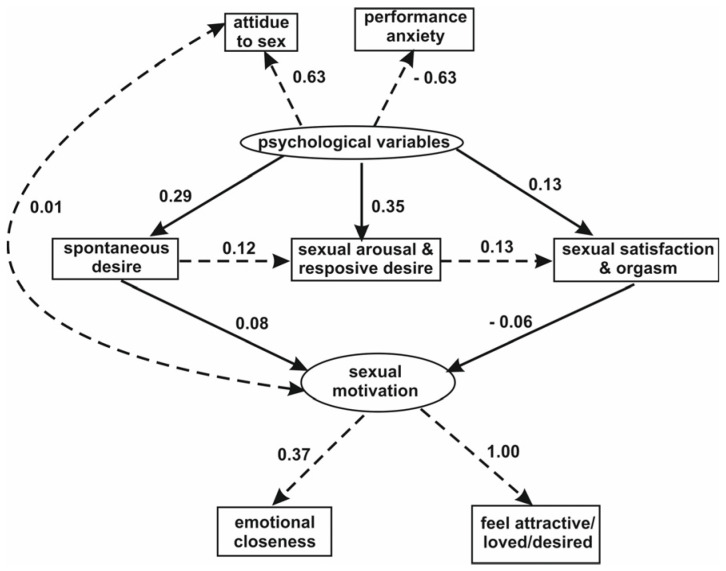
An example of a structural equation model based on Basson’s theoretical model of female sexual reactions. This model shows mutual dependences between factors producing sexual satisfaction and orgasm. The dashed lines denote the statistically insignificant correlations.

For instance, a confirmatory path analysis validated Basson’s model, describing a cycle of female sexual reactions and showing correlations between the studied variables (Figure 10) [34].

The model in Equation (1) treats the independent variables in a deterministic manner. However, because of the inter-individual variability of sexual reactions, it is reasonable to treat all of them as random variables. Thus, a complete model of human sexual reactivity can be expressed in the following form:

(2) $$P_{y}(y)=f\left(P_{x,u,d}(x,u,d)\right)$$

where $P_{y}$ is the probability distribution of sexual responses in a given population; $P_{x,u,d}$ is the joint probability distribution of the state variables, the confounders, and the stimulating variable included in the model; and f is a function linking the two probability distributions. Because the joint probability function contains a dependence relationship among the random variables, we can rewrite it in the following form:

(3) $$P_{v}(v)=\prod_{i=1}^{n+k+m}P_{v}(v_{i}|\mathrm{parent}(v_{i})),$$

where $v={\left[\begin{array}{ccc} x & u & d \end{array}\right]}^{T}$ and parent($v_{i}$) denotes the conditioning variables directed related to the $v_{i}$ element of this vector. Thus, a joint probability distribution can be factorized to the corresponding conditional probability distributions representing a structure of mutual relationship variables. Such a model is nonparametric because no form of relationship is assumed. It can be obviously proven that, if the relationships between variables are linear, model (3) could be identified using SEM methodology. Models expressed using Formula (3) are called Bayesian networks. Factorization is conducted based on measuring data in learning Bayesian net algorithms. The identified model is isomorphic, with a direct acyclic graph consisting of nodes representing model variables and direct arrows representing plausible causal relationships among the variables. Yet, such models are rarely used in sexual research; when they are used, they are usually applied to identify causal relationships between brain areas controlling sexual reactions [35]. Annika Gunst et al. applied the methodology of Bayesian networks to identify a factor structure leading to low sexual desire in women [36]. The results obtained from these models indicate that sex-related pain and body dissatisfaction may play roles in precipitating decreases in sexual desire in women.

When discussing models based on Bayesian networks, one should mention that these models describe sexual reactions at the population level, or sexual reactions of the most likely person if the learning process used the maximum likelihood estimators. Formally, inference based on a random sample drawn from a population was thought to be better than that based on a single subject because of estimator consistency and unbiasing by confounders. Unfortunately, the random draw does not ensure a uniform distribution of any confounder’s values/levels. For instance, if—in a given population—most women are in the luteal phase of their menstrual cycles, then this phase will predominate in the random sample. The phase of the menstrual cycle is a typical confounder in all models describing female sexual reactions.

Therefore, a single-subject study design is being developed. It enables the identification of models describing the sexual reactions of a given volunteer or patient. If the model identified for a given person mimics others, then we could judge that this model will sufficiently describe sexual reactions well. In this case, one can apply well-known system identification methods, such as models expressed by transfer functions, ARMAX, or NARMAX equations [37]. Moreover, in studying the dynamic sexual response to a given time-varying stimuli, we propose using the concept of linear or nonlinear Granger causality or the transfer entropy estimator to develop a possible direct acyclic graph for a given subject [38]. The models identified for several subjects can be aggregated at the population level using mixed-effect models or the Bayesian approach.

The above-mentioned models belonging to the class of “black box models” are the most frequently used in sexology because their building does not require any a priori knowledge of the studied phenomenon. On the contrary, the so-called “white box models” are based on biophysical principles, and are expressed as differential equations, which are used the least in sexology. To the best of our knowledge, such models are used for two sexual issues. At the individual level, hemodynamic models are developed to study the relationship between systemic blood pressure, cardiac output, and the rigidity of penile erection. The most validated model was presented by Yildirim et al. [39]. These authors used the classical lumped parameter model of the cardiovascular system extended to the dynamics of penile blood circulation. This model predicts penile size change during erection, with approximately a 15% error, according to clinical measurements. This shows sufficient accuracy, allowing for the optimization of hypotensive treatments regarding the quality of male sexual life. Other models are constructed at the population level. They are based on mathematical models of epidemics and describe the spread of sexually transmitted diseases. The most advanced model using stochastic differential equations was proposed by Liang et al. for a description of HIV spread amongst people who inject drugs [40]. This model proved that a smaller fraction of such people cleaning their needles, or a smaller reduction in syringe-sharing rates, would still be sufficient to eliminate disease transmission.

Additionally, we emphasize that all published models describing the mechanism of sexual reactions at different resolution levels are not sufficiently valid. Because of the high complexity of this mechanism, a model that fits the empirical data well does not warrant good predictive accuracy. Thus, the models of sexual reaction mechanisms should be validated using cross-validation procedures to assess their external validity [41]. Clinicians or researchers should also evaluate the ecological validity in participant observational studies.

Moreover, we suggest that the model fidelity should also be evaluated. The model fidelity is how much a model agrees with actual sexological knowledge. The simplifying assumptions and the granularity level cannot be contrary to this knowledge.

Finally, we postulate that the sexological ontology must be based on properly validated mathematical models with different resolutions. These models should approach the form given by Equation (1). Cooperation with biomedical engineers is essential to achieve this goal.

## 5. Devices and Systems Supporting Sex Therapy

**Figure 11:**
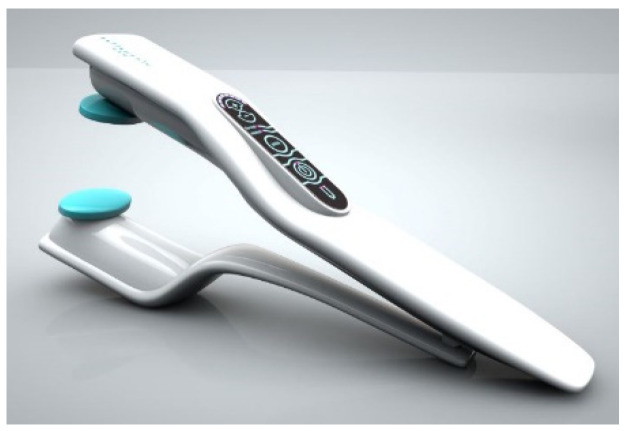
FDA-approved vibrator for the therapy of penile erectile dysfunction.

Due to the lack of a well-validated model of human sexuality, treatments of most sexual dysfunctions are still intuitive, depending on the therapeutic school. Indeed, only the pharmacological treatment of erectile dysfunction is based on the physiology of erection. However, an insufficient number of sex therapists has led to the development of numerous devices for improving sexual life. They are called “sexual toys”, but they are increasingly being used in effective sex therapy. In 2013, E. Stabile postulated that these devices should be treated as medical equipment under the FDA’s control. Moreover, some vibrators and a clitoral sucking pump have already been approved by the FDA for sexual rehabilitation (Figure 11) [42]. The postulate prompted technical studies of vibrators, which were the most popular in the USA, to estimate their amplitudes and frequencies. All of the vibrators tested generated a sinusoidal wave, with a mechanical amplitude ranging between 37 and 783 µm, and a frequency between 43 and 148 Hz [43]. It was proven that vibrators are useful in the therapy of arousal and orgasm dysfunction in women, as well as in erectile dysfunction and premature ejaculation in men [44,45]. Now, the same devices are offered for men as well as women. However, some disorders require stimulation, improving genital sensitivity by increasing innervation and congestion. In other cases, such as premature ejaculation, vibrations should desensitize the penis. Accordingly, different vibrations may be more effective in the therapy of vaginismus than in the therapy of too-relaxed pelvic floor muscles. Thus, it seems reasonable to differentiate vibrator constructions depending on their clinical purpose. Moreover, the relationship between the shape, frequency, and amplitude of the mechanical vibrations and patient age and body composition, or the activity of the sympathetic nervous system, is still unknown. Additionally, in some women, frequent use of the same vibrator may produce unwanted habituation mechanisms [46].

Therefore, a therapeutic vibrator should have vibrations with a randomly varied frequency and amplitude at various times. This should also protect against conditioning on a single tactile stimulus. Another idea can be a vibrator designed for biofeedback sexual therapy. This could be a self-tuning device consisting of a neural network and using heart rate or electrodermal signals as biofeedback. The other group of devices is used for the therapy of patients with vaginismus. A smart vaginal dilator can also be used for this purpose. Such a dilator could have tensometers measuring the tension of the pelvic floor muscles, with dilatation occurring when the patient relaxes their pelvic muscles.

**Figure 12:**
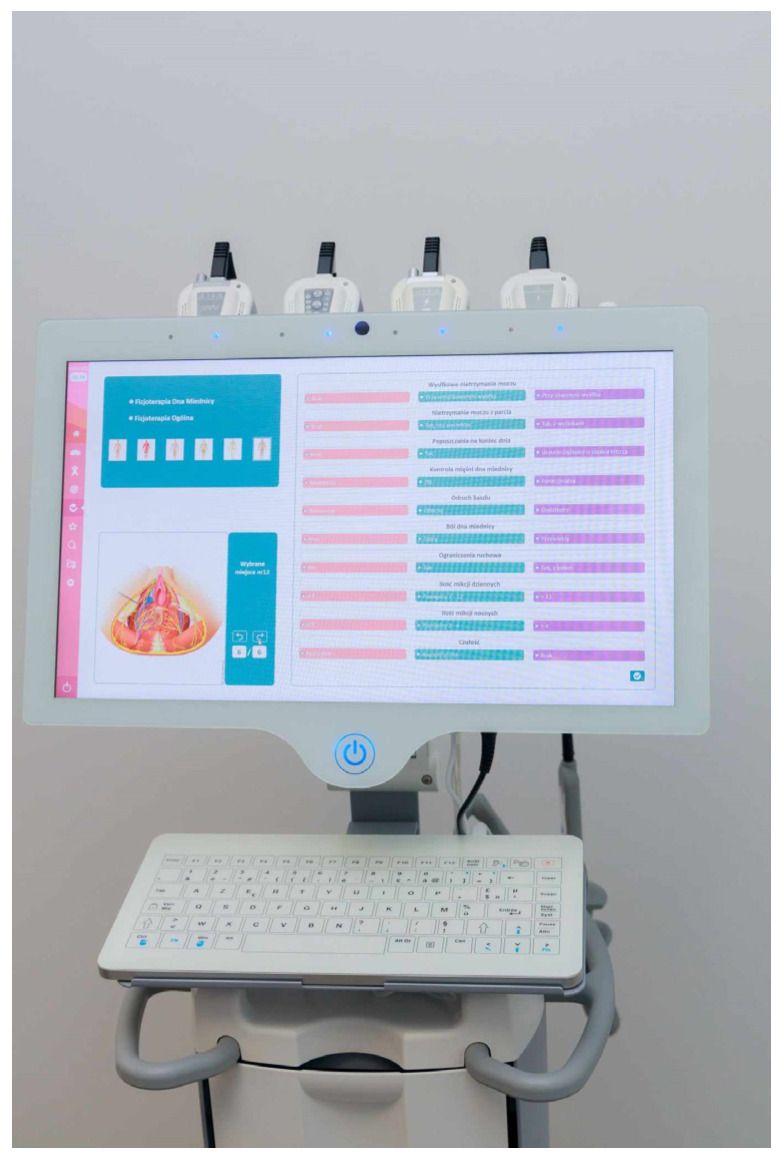
The most advanced medical devices intended for physiotherapy of pelvic floor muscles [47].

There is also more advanced equipment intended for the physiotherapy of pelvic floor muscles, e.g., Phenix Liberty New Generation (Figure 12). This combines manometrical rectal and vaginal sensors with the surface electrodes used for pelvic muscle stimulation. The software contains many therapeutic scenarios, depending on the clinical problem. There is also the possibility of adapting the stimulation pattern based on biofeedback [47].

The development of medical tools can also effectively support the sexual rehabilitation of people with disabilities. An electrical pump helping to achieve penile erection is an example of such a device. Modern pumps can be controlled using a mobile application. Electrostimulation is another solution designed for people suffering from a spinal injury. This method enables the improvement of sensory pathways via peripheral innervation, better peripheral blood circulation, and the development of new nonspecific erogenous zones. However, there is a need to determine the optimal parameters for the electrical stimuli.

**Figure 13:**
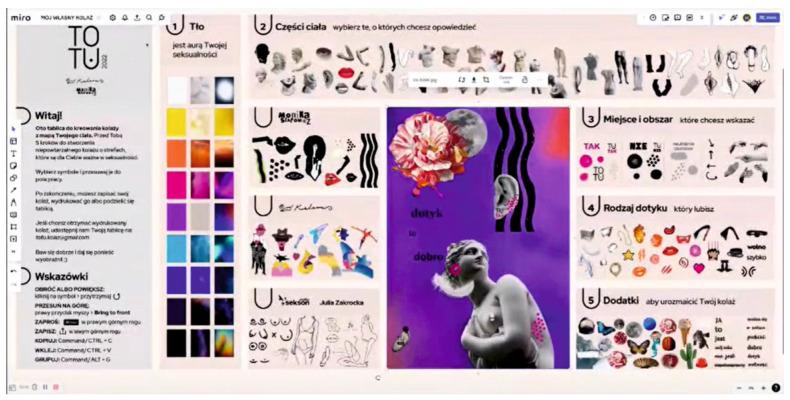
A screenshot of the ToTu application, enabling the selection of a body part, degree of pleasure (including unpleasant), type of touch, and additional individually interpreted icons (https://www.youtube.com/watch?v=evB_feTSzU8&t=21s, accessed on 16 August 2025).

We have also recently observed an increased application of information technology in sex therapy in terms of mobile applications and computer programs. An example of the former is a Polish mobile application called ToTu (Figure 13). This increases communication between partners, allowing for a mutual examination of their own erogenous zones while sending an image of their body showing pleasant, neutral, and unpleasant areas to touch [48].

There are applications available on the market with the goal of suggesting new foreplay scenarios and sexual positions or enabling the communication of emotions or the remote control of vibrator parameters to allow for a long-distance sexual relationship. Interestingly, there is also a special mobile diary application for monitoring the variability of female sexual desire and affecting factors such as sleep, alcohol, and coffee. This mobile software cooperates with a smart vaginal vibrator containing accelerometers and tensometers. The preferable vibration frequency, vibration force, and time to orgasm are presented on a graph. Then, the user can identify the relationship between the affecting factors and the mode of sexual stimulation (Lioness is the best for tracking sex drive) [49]. This app can also be very useful in sex therapy.

The latest trend in applying biomedical engineering, especially information technology, is called virtual reality exposure therapy (VRET). The most common etiological factor for sexual dysfunction is anxiety, and studies have proven the effectiveness of VRET in the treatment of anxiety disorders, depression, and posttraumatic stress disorders [50]. This method can also be useful in the therapy of vaginismus, genitourinary pain syndrome, and premature ejaculation. Additionally, mindfulness is becoming the most popular sex therapy technique. Virtual reality can be combined with mindfulness to generate relaxing images. Furthermore, virtual reality associated with an artificial vagina (a mechanical phantom) may be effective in the treatment of psychogenic masturbation-dependent erectile ejaculatory dysfunctions. Finally, VRET coupled with mechanical or electrical stimulators seems to be helpful in the therapy of arousal and orgasmic disorders, as well as in the sexual rehabilitation of people with disabilities. For this very purpose, a multitransmitter suit can be added. Due to these reasons, VRET should be treated as a modern type of behavioral therapy.

In modern psychiatry, computer game therapy could support cognitive therapy, such as VRET. Three-dimensional simulation programs may play an educational role, and computer role-play games can teach inter-partner communication and flirting methods. Such games can also develop emotional intelligence by reading game actors’ body language. It has been proven that game therapy effectively decreases the number of risky sexual contacts, as well as preventing sexually transmitted diseases [51].

## 6. Conclusions

Sexual health is one of the most important components of human well-being. However, following Janice Irvine, there is an institutionalized stigma in the production of sexual knowledge [52]. Sexuality research is perceived as “dirty” work by systematic practices of the university system. These institutional practices are shaped by cultural schemas regarding sexuality, enacted through the cognitive and affective bias of institutional actors. The construction of sexuality research as “dirty” work not only affects the researchers themselves, but also shapes the broad production of sexual knowledge.

However, effective and safe management of patients’ sexual health requires the development of evidence-based sexology. This is particularly important in the age of digital health. For this purpose, we introduced a new concept of sexological ontology as a tool for the creation of sexual knowledge. For the first time, we postulate that this ontology must be built on properly validated models describing human sexual reactions at different resolution levels, taking the biopsychosocial character of human sexuality into account. Therefore, we pointed out the necessity of perceiving the sexual reaction mechanism as a complex, hierarchical, nonlinear, and dynamic system, and proposed a dynamical model of human sexual reactions. We emphasize that this model must obviously contain the confounding variables most frequently neglected in sexological studies. It enables the identification of causal relationships between sexual stimuli, psychophysiological states, and sexual reactions or behaviors. We are also convinced that the original, biocybernetic view of human sexual reactions allows scientists and clinicians to incorporate distributed results from partial studies into the internally consistent model.

According to our concept, the model of human sexual reactions should be identified based on reliable and valid measurements. So, we presented a new review of experimental measuring methods and clinically used devices that are ethical and ensure a good quality of acquired data.

Discussing the system modeling approach to human sexuality, we first drew attention to the necessity of model validation using cross-validation procedures and assessing ecological validity. In our opinion, only the properly valid model can be incorporated into sexological ontology.

Moreover, the presented concept of sexological ontology opens a wide opportunity for the applications of biomedical engineering to a better understanding of human sexuality and safety management of sexual health at an individual and population level, using accessible information technology.

Finally, following George Box, we remember that “all models are wrong, but some are (also clinically) useful”. This is important in clinical decision-making, particularly in sexology, where clinical decision-making is an ill-posed problem because of the very limited and biased accessible information about patient sexual health. This significantly increases the risk of cognitive biases, leading to misdiagnosis and overtreatment.

## Figures and Tables

**Table 1:** Brain areas controlling sexual response, identified in fMRI studies.

Brain Area	Function
Reward system	Triggers sexual motivation
	Mate choice
Thalamus	Relays erotic stimuli incoming from the spinal cord
Hypothalamus	Coordinates autonomic events in sexual behavior
	Mate choice
Amygdala	Gives emotional significance to incoming erotic stimuli
	Mate choice
	Modulates sexual drive
Septal region	Modulates sexual drive
Prefrontal cortex	Blunts the initiation of sexual behavior
	Modulates sexual drive
Cingulate cortex	Processes sexual stimuli in conflictual contexts
	Modulates sexual drive
Insula	Awareness of tumescence of erectile organs
	Modulates sexual drive

## Data Availability

No new data were created or analyzed in this study. Data sharing is not applicable to this article.
